# Neural correlates of vicarious reward processing and peer victimization experiences in late childhood

**DOI:** 10.1016/j.dcn.2024.101499

**Published:** 2024-12-24

**Authors:** Simone Dobbelaar, Sanne Kellij, René Veenstra, Berna Güroğlu

**Affiliations:** a Developmental and Educational Psychology Department, Institute of Psychology, Leiden University, the Netherlands; b Leiden Institute for Brain and Cognition, Leiden University, the Netherlands; c Netherlands Organization for Applied Scientific Research (TNO), the Netherlands; d Department of Sociology, University of Groningen, the Netherlands

**Keywords:** Vicarious reward processing, Ventral striatum, fMRI, Victimization, Trust, Late childhood

## Abstract

This preregistered study examined the neural correlates of vicarious reward processing and subsequent trust behavior in relation to experiences of victimization averaged over two years in late childhood. This study used a sample of children with prospective longitudinal data on peer victimization over the past two years (*n*_behavioral_ = 83, 49.4 % girls, *M*_*age*_ = 10.6 years, *n*_fmri_ = 62). Participants played an fMRI vicarious reward task in which they could win or lose money for themselves and two other peers. The two other peers were experimentally manipulated to either include or exclude the participant in a Cyberball task prior to the task. Additionally, trust in the two peers was assessed using a one-shot trust game. Results revealed ventral striatum activation when winning (versus losing) for oneself, and activation in the dmPFC, vmPFC and precuneus when playing for excluders rather than for oneself. Victimization predicted decreased ventral striatum activation during personal rewards, and increased activation in the dorsomedial prefrontal cortex when playing for excluders rather than for oneself. Finally, averaged victimization was associated with increased differentiation in trust toward the including and excluding peers. Together, these findings contribute to our understanding of the social cognitions and behaviors of victims of bullying.

## Highlights

• fMRI study of vicarious reward processing and peer victimization averaged over two years in late childhood.
• VS was activated when winning for oneself, dmPFC, vmPFC and precuneus when playing for others.
• Averaged victimization predicted lower VS activity during personal wins (vs. losses).
• Averaged victimization predicted higher dmPFC activity when playing for an excluder (vs. self).
• Averaged victimization was related to greater differentiation in trust to including and excluding peers.

## 1 Introduction

The period between childhood and adolescence is important for the development of social relationships, as youth begin to spend more time with peers and increasingly value peers’ opinions (Blakemore and Mills, 2014, Crone and Dahl, 2012). Whereas positive peer interactions during adolescence are associated with feelings of belonging, reward, and resilience (Bullmore et al., 2017, Güroğlu, 2022), negative peer interactions can have long-lasting negative effects. Peer victimization is a strong case of negative peer interactions and is characterized by repeated intentionally aggressive or hurtful peer behaviors (Boulton and Hawker, 2000). Experiences of peer victimization have been associated with an increased risk of adolescent depression and negative mental health outcomes (Boulton and Hawker, 2000, Haltigan and Vaillancourt, 2014, Platt et al., 2013). Although anti-bullying programs can be effective in reducing bullying rates, a subset of victims of bullying remain victimized (Kaufman et al., 2018). Elucidating the mechanisms underlying altered social cognitions and behaviors in children who have experienced peer victimization may contribute to our understanding of how best to help victims of bullying.

A useful framework for studying the social cognitions and behaviors of victims of bullying is the social information processing (SIP) model (Crick and Dodge, 1994). The SIP model proposes that individuals go through several steps when processing social information: from attending to and registering social cues (i.e., encoding), to interpreting these cues (interpretation), and to forming a behavioral response based on someone’s goals (behavioral response). Importantly, how cues are encoded, interpreted and responded to is affected by prior memories, experiences and knowledge. Victims of bullying have been found to have negative attributional styles and high rejection sensitivity (Calvete et al., 2018, Kellij et al., 2022). That is, they have been shown to be more prone to interpret social cues in a negative way (Kellij et al., 2022).

An important sociocognitive change in the transition to early adolescence is an increase in reward sensitivity, particularly in social contexts and among peers (Crone and Dahl, 2012, Foulkes and Blakemore, 2016). This increased reward-sensitivity may underlie novelty-seeking and risk-taking behaviors in adolescence and promote learning new skills, maintaining friendships, and enhancing well-being (Crone and Dahl, 2012, Telzer, 2016). Based on the SIP model, we aimed to examine whether prior victimization experiences affected both the interpretation of social information, such as winning or losing rewards for liked and disliked others, and subsequent behavioral responses, such as trust in liked and disliked others. Therefore, the aim of this study is to examine the neural correlates of reward processing in social interactions, and to test whether peer victimization experiences averaged over two years are related to neural reward processing and trust behaviors toward peers who previously showed including or excluding behaviors.

### 1.1 Neural correlates of reward processing

To examine the neural mechanisms underlying reward sensitivity, studies often combine functional magnetic resonance imaging (fMRI) with gambling paradigms in which participants can win or lose money. These studies consistently point to the ventral striatum (VS) as a key brain region for reward processing (Delgado, 2007, Haber and Knutson, 2010) and a key region in affective processing (Nelson et al., 2005). The VS has been found to be activated not only when winning for oneself, but also when winning money for others, referred to as vicarious reward processing (Braams et al., 2014, Braams et al., 2014, Schreuders et al., 2021). More specifically, increased VS activation in adolescence has been demonstrated when winning (versus losing) for oneself, a best friend, or parents (Braams et al., 2014, Braams and Crone, 2017b, Brandner et al., 2021), but not when winning for an unknown stranger (Brandner et al., 2021). Moreover, winning for a friend resulted in greater VS activation when adolescents experienced greater closeness with that friend (Fareri et al., 2012, Schreuders et al., 2021). Furthermore, losing on behalf of a disliked individual, but not on behalf of oneself or a close friend, may lead to increased VS activation as well (Braams et al., 2014a). This suggests that negative consequences for disliked individuals may also elicit feelings of reward.

In addition to the VS, several other brain regions have been proposed to be involved in vicarious reward processing, including the temporal parietal junction (TPJ), the precuneus, and the medial prefrontal cortex (mPFC; Braams et al., 2014a; 2014b). This network of regions is often referred to as the social brain network (Blakemore, 2008), as these brain areas are often activated during perspective taking and when thinking about the thoughts and intentions of others (Blakemore, 2008, Blakemore, 2012, Carrington and Bailey, 2009, Van Overwalle, 2009, Van Overwalle and Baetens, 2009). Consistent with the other-focused functions of this network, the TPJ, precuneus and mPFC were previously found to be involved in processing rewards and losses for others, but not for the self (Braams et al., 2014a). During late childhood and early adolescence, this network of brain regions is particularly active, which may reflect the increased salience of peers and social belonging during this developmental period (Blakemore and Mills, 2014, Somerville, 2013). Therefore, in this study, we will examine the involvement of both the VS and the TPJ, precuneus and mPFC in vicarious reward processing in late childhood.

### 1.2 Peer victimization and reward processing

Vicarious reward processing appears to be modulated by the social context and prior social experience with a person (Bhanji and Delgado, 2014), in line with the SIP framework. A history of negative peer experiences may alter the interpretation of social information, such as neural responses in the reward system. For example, adolescents who were less accepted by their peers showed more VS activation when they won money for themselves than adolescents who were more accepted by their peers (Meuwese et al., 2018). Very low and high levels of peer adversity have been linked to increased functional connectivity between the VS and dorsolateral prefrontal cortex and blunted functional connectivity between the VS and posterior cingulate cortex when receiving rewards (Rudolph et al., 2021). In addition, peer victimization has also been associated with reduced mPFC activation during reward processing (Casement et al., 2014), and in highly victimized adolescents, greater wariness has been related to greater amygdala activation when processing unpredictable positive feedback (Jarcho et al., 2019). However, whether victimization is associated with altered neural vicarious reward processing, when winning or losing for others, has not been previously examined. Victimization has been associated with increased sensitivity to social stimuli, as reflected in increased rejection sensitivity (Cubillo, 2022, Gao et al., 2019, Kellij et al., 2022) and increased social monitoring (Telzer et al., 2020). Potentially, this rejection sensitivity may be reflected in less rewarding feelings when winning money for disliked peers, such that children with more peer victimization experiences show less VS activation when winning and more VS activation when losing for disliked peers. The second aim of this study is to examine the relation between neural vicarious reward sensitivity (i.e., the interpretation step in the SIP model) and peer victimization.

### 1.3 Peer victimization and trust behavior

Understanding how victims process rewards for others may help explain subsequent behavioral responses toward peers, such as trusting others, which is a key factor in the development of social relationships (Crone et al., 2022, Crone and Dahl, 2012, Lahno, 1995). Neural activation in reward-related areas has been reported during trust decisions and depends on prior experience with the trustee (Bellucci et al., 2017, Bhanji and Delgado, 2014, Delgado et al., 2005, Fareri et al., 2012). Neural activation during vicarious reward processing has been associated with prosocial behavior (Contreras-Huerta et al., 2023, Morelli et al., 2018), suggesting a link between reward sensitivity and subsequent behavior. Striatum activation during unexpected social feedback has also been associated with decreased trust (Poore et al., 2012), and increased striatum activation during positive rewards in a trust game has been linked to prediction error learning of trustworthiness (Fareri et al., 2012b). Victimized and chronically rejected adolescents share less with unfamiliar others (Will et al., 2018), show less prosocial behavior toward peers (Rappaport et al., 2019), and have lower trust learning (Lenow et al., 2015) compared to non-victimized adolescents. Therefore, the final goal of this study was to examine how neural vicarious reward processing and peer victimization are related to trust behaviors toward others (i.e., the behavioral response step in the SIP model).

### 1.4 The current study

This preregistered study had several aims (see Dobbelaar et al., 2024). First, we examined the neural correlates of vicarious reward processing for peers who have recently engaged in including (i.e., liked peers) or excluding (i.e., disliked peers) behavior. We hypothesized that winning for oneself and for an includer would result in greater VS activation than winning for an excluder (hypothesis 1a), that losing for an excluder would result in greater VS activation than losing for an includer and for oneself (hypothesis 1b), and that VS activation when winning (vs. losing) for an includer and an excluder would be positively correlated with self-reported pleasure of winning for an includer and excluder, respectively (hypothesis 1c). In addition to VS activation, we explored the involvement of the dmPFC, vmPFC, precuneus, and TPJ in secondary analyses.

Our second aim was to examine the effects of prior peer victimization on vicarious reward processing (i.e., the interpretation of social information). We hypothesized that VS activation to winning versus losing for oneself would be higher for children with higher victimization scores than for children with lower victimization scores (hypothesis 2a), that VS activation to winning versus losing for an excluder would be lower for children with higher victimization scores than for children with lower victimization scores (hypothesis 2b), and that the difference in VS activation between winning (vs losing) for oneself, and winning (vs losing) for an excluder would be greater for children with higher victimization scores (hypothesis 2c).

Finally, we tested whether vicarious reward processing and peer victimization were related to subsequent trust behaviors (i.e., the behavioral response to social information). We hypothesized that participants with greater VS activation when winning for the includer and the excluder would show more trust behaviors toward the includer and the excluder (hypothesis 3), that participants would trust the includer more than the excluder (hypothesis 4a) and that children with higher victimization scores would trust less than children with lower victimization scores, regardless of the target (hypothesis 4b).

## 2 Methods

### 2.1 Participants

We used data from 83 children who participated in the Social Cognition and Attention Regarding victimS (SCARS) project (49.4 % girls, age = 10.63 ± 1.00 years; see https://osf.io/vnxtq for an overview of the larger project). Participants were recruited through schools participating in the KiVa anti-bullying program. As part of this program, self-report measures of peer victimization were collected in the years prior to the study. Participants were included in SCARS if they had data on at least two self-reports of the victimization questionnaire in the two years prior to the study, did not have epilepsy, were not taking psychotropic medications that could not be stopped for 24 hours, and did not have MRI contraindications (e.g., braces).

Recruitment took place in the winter of 2020–2021 by contacting 152 KiVa schools within a radius of 100 kilometers from the Leiden University Medical Center in Leiden. Recruitment partially overlapped with the second lockdown of the Covid-19 pandemic in the Netherlands (December 2020 – February 2021). Therefore, instead of visiting schools during the recruitment process, schools were asked to send a letter and a short introductory video to the parents of children in Dutch grades 6–8 (including children aged 8–12). Forty-three schools agreed to participate, 104 schools did not, and 5 schools could not be reached. Of the 43 schools that sent the letter, 156 parents responded and gave their consent to participate and to access their child's data on peer victimization as part of the KiVa project. Children who had at least two self-reports of victimization in the two years prior to the study were invited to participate, resulting in a total sample size of 83 children (from 23 schools). Of these 83 participants, 21 participants were excluded from the MRI sample due to lack of MRI data due to anxiety (*n* = 4), incomplete MRI data (*n* = 2), processing errors (*n* = 1), and motion (*n* = 14). Table 1 provides the demographic characteristics of the total sample (*n* = 83) and the MRI sample (*n* = 62). Participants with MRI data (*n* = 63) were older, *t*(30.56) = 2.71, *p* = .011, and reported lower averaged victimization scores, *t*(28.61) = -2.29, *p* = .030, than children without MRI data (*n* = 21).

**Table 1:** Demographical characteristics of the total sample and MRI sample.

	Total sample (*n* = 83)	MRI sample (*n* = 62)
Age in years (*M±SD*)	10.63 ± 1.00	10.81 ± 0.92
Age range in years	7.9 – 12.8	8.7 – 12.8
Girls (%)	49.4	50.0
Psychiatric diagnosis (n)	8	6
– *ADHD/ADD*	2	2
– *Autism spectrum disorder*	2	2
– *Anxiety disorder*	1	1
– *Dyslexia*	3	1
Parental education (n (%))		
– *Both parents master’s degree or higher*	24 (28.9 %)	17 (27.4 %)
– *Both parents at least bachelor’s degree*	28 (33.7 %)	21 (33.9 %)
– *Both parents at least senior general track in secondary/tertiary vocational training*	20 (24.1 %)	16 (25.8 %)
– *Both parents at least vocational track of secondary school or first three years of senior general track in secondary school*	11 (13.3 %)	8 (12.9 %)
Victimization averaged over two years (*M±SD*)	1.43 ± 0.47	1.36 ± 0.42

The study was approved by the Medical Ethical Committee Leiden Den Haag Delft (NL71576.058.19). The data included in this study were collected from January 2021 to March 2022. During this period, there were two lockdowns in the Netherlands, which included primary school closures (December 2020–February 2021, and December 2021–January 2022). Only the first four participants participated during a lockdown period, while the reopening of schools had already been announced.

## 3 Procedure

Data collection took place during laboratory visits at the Leiden University Medical Center. First, participants were informed about the laboratory visit, and parents and 12-year-old participants (*n* = 5) signed an informed consent form. Participants were familiarized with the scanning procedure using a mock scanner and practiced the MRI tasks. They then participated in the scanning procedure, which consisted of a structural scan and three fMRI tasks (i.e., an emotion recognition task (Kellij et al., 2024b), a Cyberball task (Kellij et al., 2024a) and a vicarious reward task). Participants then completed behavioral tasks and questionnaires. The lab visit lasted approximately three hours in total. As compensation, participants received a goodie bag, financial compensation of 50 euros, and reimbursement for travel expenses.

## 4 Measures

### 4.1 Victimization

Victimization was measured using the Olweus Bully/Victim Questionnaire (Olweus, 1996), which consisted of six items. First, participants were given the definition of bullying (“Bullying is when some children repeatedly harass another child. Thus, bullying is that you are mean to someone else over and over again. It is difficult for the child who gets bullied to defend themselves against it.”). This was followed by a global question on victimization (“How often have you been bullied since the last questionnaire / during the last couple of months?”). The following five questions focused on the number of bullying experiences for specific types of victimization (i.e., verbal, exclusionary, physical, relational, and online). All questions were answered on a 5-point Likert scale (1: not at all; 2: once or twice; 3: two or three times per month; 4: about once a week; 5: several times per week).

In the two years prior to the laboratory visit, participants reported their victimization twice a year as part of the KiVa anti-bullying program. Participants with at least two self-reports on the victimization questionnaire were included in this study (*n* = 83, 41.0 %: two self-reports, 47.0 %: three self-reports, 9.6 %: four self-reports, 2.4 %: 5 self-reports). Victimization was also assessed during the laboratory visit.

At each time point, victimization was calculated by averaging the six victimization items. To measure averaged victimization, we averaged the victimization scores from all available time points per individual (i.e., scores from both the years prior to the laboratory visit and from the laboratory visit; see https://osf.io/x32g9 for validation of this method).

### 4.2 Cyberball task

**Fig. 1:**
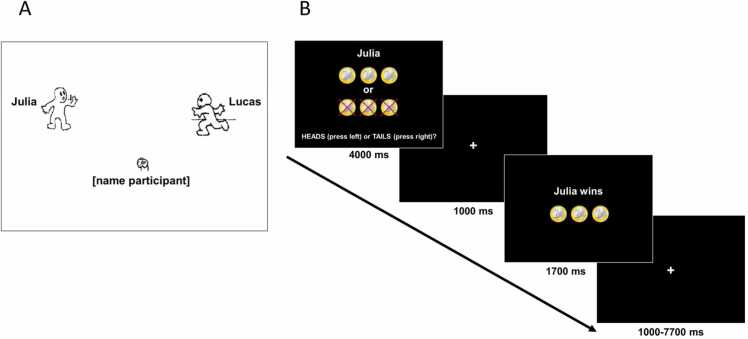
Experimental tasks. A) The Cyberball task. B) Schematic representation of a trial in the vicarious reward task.

In the fMRI procedure, a Cyberball task was administered prior to the vicarious reward task (Kellij et al., 2024a). The Cyberball task is a virtual ball-tossing game involving the participant and two unfamiliar players (Williams et al., 2000; see Fig. 1a). When participants received the ball, they could choose to toss the ball to one of the other two players. The Cyberball task consisted of an inclusion and an exclusion block of 30 trials each. In the inclusion block, participants played with two preprogrammed other players (a boy and a girl), whose names were displayed above the players. In this block, each player received the ball an equal number of times. Next, in the exclusion block, the participant played with two other preprogrammed players (a boy and a girl), depicted with two new names. In this block, the participant received the ball once, but was excluded by the other players for the remainder of the block. The names used for the players were counterbalanced across participants. Participants were instructed that they would play the game online with real other children, and that they would play the two blocks of the Cyberball game with different children. After the MRI procedure and exit questions, participants were debriefed about the fact that the other players were not real. On average, participants felt more included, reported higher need satisfaction and had a more positive mood after the inclusion block compared to the exclusion block (Kellij et al., 2024a).

### 4.3 Vicarious reward task

A previously validated gambling task was used to measure neural activity during vicarious reward processing (Braams et al., 2014, Braams et al., 2014, Braams and Crone, 2017b, Schreuders et al., 2021). In this task, participants chose heads or tails and then received feedback about whether they had won or lost money. Participants were instructed that they were playing the game for themselves, the same-sex peer from the inclusion block in the Cyberball task (i.e., the includer), and the same-sex peer from the exclusion block in the Cyberball task (i.e., the excluder).

The vicarious reward task consisted of two blocks of 36 trials (50 % win trials and 50 % loss trials). Each trial consisted of a trial onset screen indicating who participants were playing for (self, the includer, or the excluder) and requiring participants to indicate whether they would pick heads or tails (4000 ms), followed by a fixation screen (1000 ms), a feedback screen indicating whether participants had won or lost money (1700 ms), and a variable intertrial jitter (1000–7700 ms, see Fig. 1b). Trial order and timing were optimized for fMRI analysis using OptSeq (Dale, 1999). Participants played 24 trials for each of the three targets. In addition, three different coin distributions were included to ensure task engagement: winning two coins or losing five coins, winning three coins or losing three coins, and winning five coins or losing two coins. To increase statistical power, these distributions were not analyzed separately (see Braams et al., 2014a, 2014b).

Participants were instructed and familiarized with the task during the practice session. They were told that the coins won during the task would translate into a real monetary reward at the end of the experiment. Unknown to the participants, the total reward of the task was not related to the amount of money won during the task, but was a set amount. Participants received an additional 3 euros for the vicarious reward task and the trust game (along with the 50 euros financial compensation for the laboratory visit).

### 4.4 Trust game

After the fMRI procedure (including the Cyberball game and the vicarious reward task), participants played a one-shot trust game (Berg et al., 1995) outside the scanner on a computer with the four players from the Cyberball game (i.e., two includers and two excluders; two of whom they also played the vicarious reward task with). Participants were instructed to choose whether to divide ten coins equally between themselves and the other player (5:5 coins and the game ends) or to give all the coins to the other player. In the latter case, the total number of coins would be tripled to 30 coins, and the other player could choose to split the coins equally (15:15 coins) or take all the coins themselves (30 coins). Thus, choosing the second option reflects trust with a potentially higher outcome for the participant. If participants chose to trust the other player, they received no feedback on the other player’s choice. Participants were told that one coin represented one euro, and that at the end of the visit one of the choices would be paid out to the participant. At the end of the visit, participants were not informed of the specific outcome of the trust game, but were only told of the total amount earned from both the trust game and the vicarious reward task combined. Trusting behavior for the includers and excluders was defined as a binary variable (no trust = option 5:5 coins; trust = option to give coins to other player) for the specific includer and excluder who were also the targets in the vicarious reward task.

### 4.5 Self-reported pleasure and exit questions

After the fMRI procedure and the trust game (outside the scanner), participants answered exit questions about the targets they played for during the tasks. To measure self-reported pleasure, participants were asked to indicate how much they enjoyed winning and losing separately for the includer and the excluder (Braams and Crone, 2017b). In the other exit questions, which served as a manipulation check, participants were asked to indicate who they played for in the scanner, how much they liked each player, how much they thought each player deserved to win, and how much they thought each player thought the participant deserved to win. Responses were provided on a 10-point Likert scale ranging from 1 ('not at all') to 10 ('very much').

## 5 fMRI analyses

### 5.1 fMRI data acquisition

MRI data were acquired on a 3.0 Tesla Philips Achieva TX scanner, using a 32-channel standard whole-head coil. To minimize head motion, foam inserts were added within the head coil. The experimental tasks were displayed on a screen placed behind the scanner, that could be viewed through a mirror on the head coil. Functional MRI scans of the vicarious reward task were acquired during two functional runs using a T2 * -weighted echo-planar imaging (EPI). The first two volumes were discarded to allow for equilibration of T1 saturation effects [field of view (FOV) = 220 (anterior-posterior; a-p) x 220 (right-left; r-l) x 120.72 (foot-head; f-h) mm; repetition time (TR) = 2.2 s; echo time (TE) = 30ms; flip angle (FA) = 80°; sequential acquisition; 40 slices; voxel size = 2.75 × 2.75 × 2.75 mm]. In addition, a high-resolution 3D T1 scan was acquired as an anatomical reference [FOV= 250(a-p) x 195.83(r-l) x 170.50(f-h) mm; TR = 7.9ms; TE = 3.5ms; FA = 8°; 155 slices; voxel size = 1.04 × 1.04 × 1.10 mm].

### 5.2 fMRI preprocessing

The fMRI scans were analyzed using SPM12 (Wellcome Department of Cognitive Neurology, London). The preprocessing pipeline included the following steps: correction for slice timing acquisition and rigid body motion, coregistration, spatial normalization to T1 templates (based on MNI-305 stereotaxic space (Cocosco et al., 1997) using 12-parameter affine transform mapping and non-linear transformation with cosine basis functions, resampling of volumes to 3 × 3 × 3mm voxels, and spatial smoothing using a 6 mm full-width-at-half-maximum isotropic Gaussian kernel. Translational movement parameters were calculated for each participant. Data of participants were excluded from the neuroimaging analyses if participants moved more than 3 mm in any volume in any direction (x,y,z) in one or more runs of the vicarious reward task.

### 5.3 First-level analyses

Individual participant’s fMRI data were analyzed using a general linear model in SPM12. Data were modeled as a zero-duration event at the onset of the feedback delivery (i.e., winning or losing). Six regressors were defined for either winning or losing for oneself, the includer and the excluder (‘SelfWin’, ‘SelfLose’, ‘IncluderWin’, ‘IncluderLose’, ‘ExcluderWin’, ‘ExcluderLose’). Trials on which participants did not respond within 4000 ms (i.e., on whether they picked heads or tails) were marked as invalid and excluded from analysis. Six motion regressors were included as covariates of no interest. The least-squares parameter estimates (PE) of the height of the best-fitting canonical hemodynamic response function for each condition were used in pairwise contrasts. Subject-specific contrast images were used in the second-level analyses.

### 5.4 Second-level (whole-brain) analyses

To examine the neural correlates of vicarious reward processing, we computed a 2 (outcome: winning or losing) x 3 (target: self, includer, excluder) repeated measures ANOVA in SPM12. In addition to the main effects of outcome and target, we explored the following contrasts, in line with our hypotheses: ‘SelfWin > SelfLose’, ‘IncluderWin > IncluderLose’, and ‘ExcluderWin > ExcluderLose’. All results were family-wise error (FWE) cluster-level corrected (*p*_FWEcc_<.05) with an initial voxel-wise threshold of *p* < .001 (uncorrected). Tables S1 and S2 report the results of the whole-brain analyses. Unthresholded statistical maps of the whole-brain contrasts are available on Neurovault (Gorgolewski et al., 2015) via: https://neurovault.org/collections/CPXVDLSJ/.

### 5.5 Region of interest analyses

**Fig. 2:**
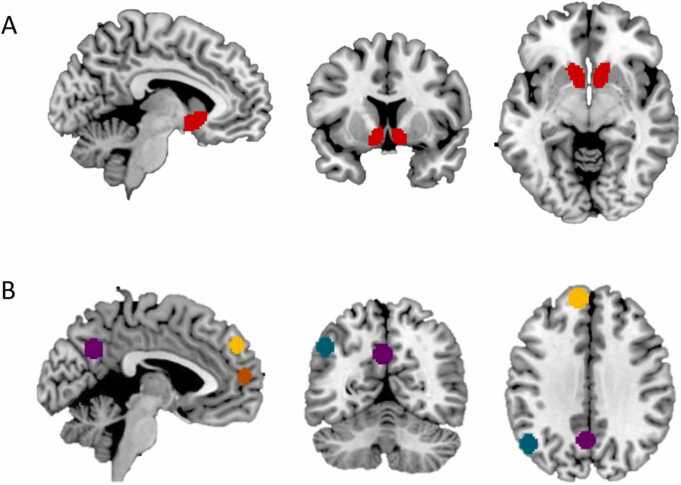
Brain regions of interest. A) ventral striatum (red). B) temporal parietal junction (green), precuneus (purple), dorsomedial prefrontal cortex (yellow) and ventromedial prefrontal cortex (orange).

In addition to whole-brain analyses, we selected regions of interest (ROIs) to examine neural activation in regions that were previously associated with vicarious reward processing. We selected the structural ventral striatum based on its role in reward-related processes and activation when winning (vs. losing) for oneself and a friend compared to an unliked other (Braams et al., 2014a; see Fig. 2a). In addition, Braams et al. (2014a) showed that the left TPJ, precuneus and dorsal mPFC (dmPFC) were more activated when receiving outcomes for others compared to outcomes for oneself. Similarly, activation in the ventral mPFC (vmPFC) has been consistently reported when thinking about others (Braams and Crone, 2017a, Van Overwalle and Baetens, 2009). The following independent ROIs were selected for secondary analyses: the left TPJ (-48,-63,39), the precuneus (-3,-60,33) and the dorsal mPFC (-9,51,36; from the main effect of Person in the Person x Outcome ANOVA during the onset of feedback, with more activation in the contrasts Friend > Self and Antagonist > Self in Braams et al., 2014a), and the ventral mPFC (1,57,12; based on an independent meta-analysis of mentalizing by Van Overwalle and Baetens 2009, as reported in Braams and Crone 2017a). Using the Marsbar ROI toolbox, 8 mm spheres were created around the reported peak coordinates (see Fig. 2b).

## 6 Statistical analyses

Statistical analyses were preregistered in the Open Science Framework (see Dobbelaar et al., 2024). Analyses were performed in R (version 4.3.0; R Core Team, 2023). Outliers were defined as *Z*-scores below −3.29 or above 3.29 and were winsorized in the analyses (Tabachnick et al., 2007). To correct for multiple testing in our analyses, we used the Benjamini-Hochberg (B-H) correction over all tests in the preregistered primary and secondary analyses. We report the B-H-corrected *p*-values in Tables S3 and S4, and in the results section when the significance of the results changes as a result of the correction.

### 6.1 Primary analyses

**Neural correlates of vicarious reward processing.** To test for outcome and target effects on VS activation (hypothesis 1a and 1b), we performed a 3 (target: self, includer, excluder) x 2 (outcome: win, lose) repeated measures ANOVA on VS activation in ROI analyses. Significant main and interaction effects were further explored using Bonferroni-corrected post hoc tests. In addition, to test whether VS activation during winning for includers and excluders relates to pleasure ratings (hypothesis 1c), we performed two regression analyses, one with ROI activation in the VS during winning (vs. losing) for the includer as a predictor and pleasure ratings of winning (vs. losing) for the includer as the outcome, and one with ROI activation in the VS during winning (vs. losing) for the excluder as predictor and pleasure ratings of winning (vs. losing) for an excluder as the outcome.

**Neural correlates of vicarious reward processing and peer victimization.** Second, to test whether victimization experiences averaged over two years were related to ventral striatum activation of winning and losing for oneself, an includer and an excluder, we conducted separate regression analyses with averaged victimization as predictor and neural VS activation as outcome variable. Specifically, we performed a regression analysis on VS (ROI) activation on the contrasts “SelfWin–SelfLose” (hypothesis 2a) and “ExcluderWin–ExcluderLose” (hypothesis 2b). In addition, we performed a regression analysis on VS (ROI) activation in the contrast “(SelfWin–SelfLose) – (ExcluderWin–ExcluderLose)” (hypothesis 2c). Exploratively, we also performed regression analyses on VS (ROI) activation in the contrast “IncluderWin – IncluderLose” and “(IncluderWin–IncluderLose) – (ExcluderWin–ExcluderLose)”.

**Neural correlates of vicarious reward processing and trust.** Third, to test whether VS activation during winning for includers and excluders predicted subsequent trust behavior (hypothesis 3), we conducted two logistic regression analyses: one with VS activation during winning (vs. losing) for the includer (“IncluderWin–IncluderLose”) as predictor and trust toward the includer as the outcome; and one with VS activation during winning (vs. losing) for the excluder (“ExcluderWin–ExcluderLose”) as predictor and trust toward the excluder as the outcome.

**Trust and peer victimization.** Fourth, we examined whether peer victimization averaged over two years was related to trust behaviors (hypothesis 4a and 4b). We conducted a logistic regression, with target (includer, excluder) and averaged peer victimization as predictors and trust behaviors (binomial) as the outcome.

### 6.2 Secondary analyses

In secondary analyses, we examined whether the TPJ, precuneus, dmPFC and vmPFC were more activated in the includer and excluder conditions compared to the self condition, regardless of the target (Braams et al., 2014a). We conducted four 2 (outcome: winning, losing) x 3 (target: self, includer, excluder) repeated measures ANOVAs with TPJ, precuneus, dmPFC and vmPFC (ROI) activation as separate outcome variables. For significant outcome, target, or interaction effects in these regions, we examined associations with averaged peer victimization in subsequent regression analyses.

We conducted two exploratory, non-preregistered analyses. First, we additionally examined whether peer victimization was related to neural activation during winning vs. losing in a social context in the TPJ, precuneus, dmPFC and vmPFC. Therefore, in separate regression analyses, we explored whether victimization predicted activation in these four ROIs during winning vs. losing for each target (i.e., “SelfWin-SelfLose”, “IncluderWin-IncluderLose”, “ExcluderWin-ExcluderLose”). These analyses are reported in Table S5. Second, we examined associations between neural activation in the TPJ, precuneus, dmPFC and vmPFC (in the contrasts with significant target or interaction effects) and trust in logistic regression analyses.

## 7 Results

### 7.1 Manipulation check

Participants reported liking the includer (*M* = 7.22 ± 2.41) more than the excluder (*M* = 4.16 ± 2.59), thinking the includer deserved to win more (*M* = 7.49 ± 2.41) than the excluder (*M* = 4.94 ± 2.71), and thinking that the includer believed the participant deserved to win more (*M* = 7.24 ± 2.22) than the excluder did (*M* = 4.39 ± 2.83; all *p*’s < .001).

### 7.2 Neural correlates of vicarious reward processing

Fig. S1 and Tables S1 and S2 present the results of the whole-brain analyses. Overlap between whole-brain activation and independent ROIs is presented in Fig. S2.

In the preregistered ROI analyses, we examined target and outcome effects in predefined ROIs using repeated measures ANOVAs with two within-person factors: target (three levels: self, includer, excluder) and outcome (two levels: win, lose). Table S3 presents the results of the repeated measures ANOVAs.

**Fig. 3:**
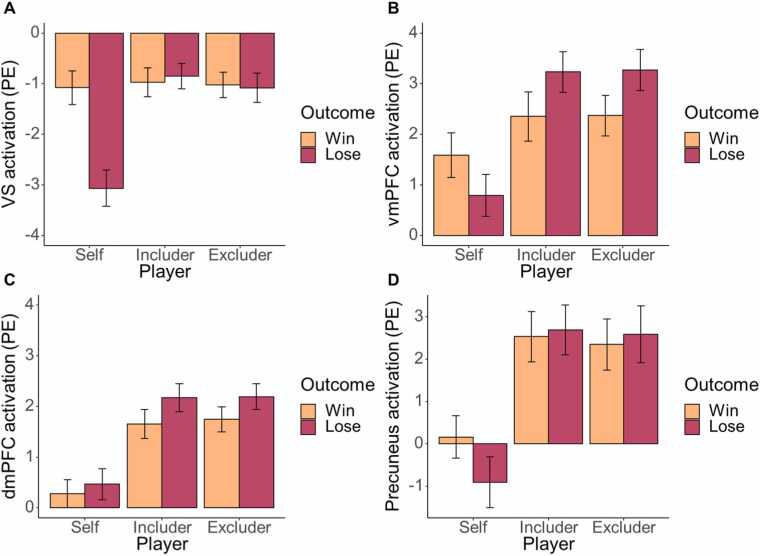
Player (self, includer, excluder) and outcome (win, lose) effects on neural activation (parameter estimates) in four regions of interest: A) ventral striatum; B) ventral medial prefrontal cortex; C) dorsomedial prefrontal cortex; D) precuneus. Error bars represent standard errors.

**Ventral striatum.** ROI analyses on VS activation showed a main effect of target, *F*(1.77,107.86) = 8.43, *p* < .001, indicating more activation during excluder and includer trials compared to self trials. Second, there was a main effect of outcome, *F*(1,61) = 6.81, *p* = .011, indicating more activation during win versus loss trials. Third, the results showed an interaction effect of target × outcome, *F*(2,122) = 12.36, *p* < .001. Bonferroni-corrected post-hoc tests revealed more VS activation during winning than during losing in the self-condition (*p* < .001), but not in the includer or excluder conditions (both *p* = 1). Furthermore, VS activation was higher when losing for the excluder and the includer, compared to losing for oneself (both *p* < .001), whereas VS activation did not differ significantly when winning for the three different targets (*p* = 1, Fig. 3a).

**vmPFC, dmPFC, TPJ and precuneus.** In secondary analyses, we examined target and outcome effects using repeated measures ANOVAs in four predefined ROIs: vmPFC, dmPFC, TPJ and precuneus. In line with the whole-brain analyses, we found effects in the vmPFC, dmPFC and precuneus, but not in the TPJ.

In the vmPFC, there was a main effect of target, *F*(2,122) = 7.01, *p* = .001, such that vmPFC activation was increased in the excluder (*p* = .001) and includer (*p* = .005) conditions compared to the self condition. In addition, there was an interaction effect of target × outcome, *F*(2122) = 3.23, *p* = .043: vmPFC activation was increased when losing for the excluder compared to for oneself (*p* = .001), and when losing for the includer compared to for oneself (*p* = .002; see Fig. 3b), whereas vmPFC activation did not differ when winning for the three targets (all *p* > .573). However, the interaction effect did not survive B-H correction, *p* = .106.

In the dmPFC, results revealed a main effect of target, *F*(1.82,111.03) = 19.52, *p* < .001, indicating increased dmPFC activation during the excluder and includer conditions, compared to the self condition (both *p* < .001; see Fig. 3c). In addition, there was a main effect of outcome, *F*(1,61) = 5.87, *p* = .018, indicating increased dmPFC activation when losing compared to winning.

In the precuneus, results revealed a main effect of target, *F*(2,122) = 14.70, *p* < .001, indicating more precuneus activation during the excluder and includer conditions compared to the self condition (both *p* < .001; see Fig. 3d).

In the TPJ, we found neither main effects nor an interaction effect of target and outcome (all *p* ≥ .075).

#### 7.2.1 VS activation and pleasure ratings of winning

Participants liked winning more for the includer than for the excluder (*t*(81) = 6.08, *p* < .001; *M*_*incl*_= 6.82 ± 2.51; *M*_*excl*_= 4.71 ± 2.70), and liked losing more for the excluder than for the includer (*t*(80) = 3.44, *p* < .001; *M*_*incl*_= 3.81 ± 2.42; *M*_*excl*_= 5.06 ± 2.78). In addition, participants liked winning for the includer more than losing for the includer, *t*(80) = 6.75, *p* < .001, but did not differ in their pleasure ratings of winning versus losing for the excluder, *t*(81) = -0.68, *p* = .500.

VS activation during winning (vs. losing) for the includer did not predict pleasure ratings of winning (vs. losing) for the includer, *t*(59) = 0.18, *p* = .856. VS activation during winning (vs. losing) for the excluder did also not predict pleasure ratings of winning (vs. losing) for the excluder, *t*(60) = -0.20, *p* = .841.

### 7.3 Neural correlates of vicarious reward processing and peer victimization

Next, we examined whether neural correlates of vicarious reward processing were related to averaged peer victimization. Table S4 presents the results of the regression analyses.

#### 7.3.1 Ventral striatum

**Fig. 4:**
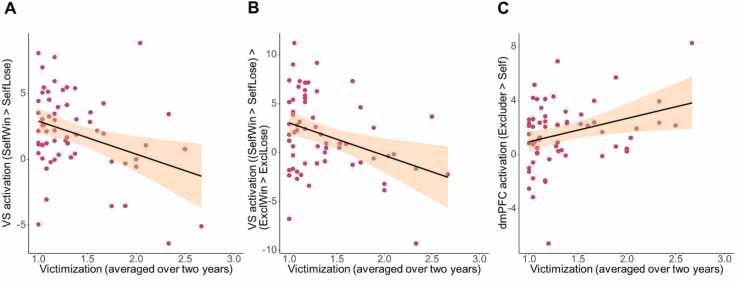
Relation between victimization scores averaged over two years and A) ventral striatum activation in the contrast ‘SelfWin–SelfLose’; B) ventral striatum activation in the contrast ‘SelfWin-SelfLose > ExclWin-ExclLose’; C) dmPFC activation in the contrast ‘Excluder–Self’.

Peer victimization negatively predicted VS activation in the “SelfWin–SelfLose” contrast (*β* = -0.34, *t*(60) = -2.82, *p* = .007), such that children with higher averaged victimization scores showed less VS activation when winning (vs. losing) for themselves compared to children with lower victimization scores (see Fig. 4a). Similarly, peer victimization negatively predicted VS activation in the “SelfWin-SelfLose – ExcluderWin-ExcluderLose” contrast (*β* = -0.35, *t*(60) = -2.88, *p* = .006), such that children with higher averaged victimization scores showed less VS activation when winning for oneself compared to winning for the excluder. Peer victimization did not predict VS activation when winning (vs losing) for the excluder, includer, or the difference between winning for the includer and excluder (all *p* ≥ .093).

#### 7.3.2 vmPFC, dmPFC and precuneus

For the vmPFC, dmPFC and precuneus, we explored whether activation was predicted by peer victimization. For each region, we focused on the specific contrasts in which target and/or outcome effects were found.

For the vmPFC, we explored whether averaged victimization predicted activation in the contrasts “Excluder–Self” and “Includer–Self”. Victimization did not predict activation in any of the contrasts (both *p* ≥ .165).

For the dmPFC, we explored whether averaged victimization predicted activation in the contrasts “Excluder–Self”, “Includer–Self”, and “Lose–Win”. Victimization was positively related to dmPFC activation in the “Excluder–Self” contrast (*β* = 0.30, *t*(60) = 2.46, *p* = .017), such that children with higher peer victimization over the previous two years showed more dmPFC activation when playing for an excluder (vs. playing for oneself) compared to children with lower peer victimization scores (see Fig. 4b). Averaged victimization did not predict dmPFC activation in the contrasts “Includer–Self” and “Lose–Win” (both *p* ≥ .056).

For the precuneus, we explored whether averaged victimization predicted activation in the contrasts “Excluder–Self” and “Includer–Self”. Victimization did not predict activation in any of the contrasts (both *p* ≥ .456).

### 7.4 Neural correlates of vicarious reward processing and trust behaviors

Next, we assessed whether VS activation upon winning (vs. losing) for the includer and excluder would predict subsequent trust toward the includer and excluder, respectively. We first compared the proportion of trust toward the different targets. The likelihood of trusting the includer did not differ from the likelihood of trusting the excluder, log(OR) = 0.21, *Z* = 0.64, *p* = .521. Results revealed no associations between VS activation and trust in either the includer or the excluder (both *p* ≥ .162).

Exploratively, we examined whether trust was predicted by social brain activation, focusing on the specific contrasts in which target effects were found. Activation in the vmPFC, dmPFC and precuneus did not predict trust in either the includer or the excluder (all *p* > .356).

### 7.5 Peer victimization and trust

**Fig. 5:**
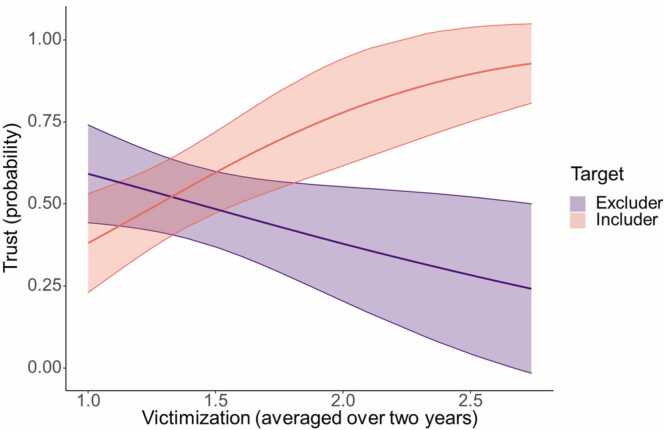
Association between averaged victimization over two years and probability of trust choices for the excluder (in purple) and includer (in orange).

Finally, we examined whether averaged peer victimization predicted trust toward the includer and the excluder in a logistic regression with target and victimization as predictors. There was a significant interaction effect of target × victimization (log(OR) = 2.62, *p* = .001, 95CI[1.10,4.33]), such that children with higher victimization scores differentiated more in trusting the includer and excluder compared to children with lower victimization scores (see Fig. 5). Post-hoc testing revealed that the likelihood of trusting the includer increased for children with higher victimization scores (log(OR) = 1.75, *p* = .006, 95CI[0.49,3.01]), whereas the probability of trusting the excluder did not change significantly with victimization scores (log(OR) = -0.87, *p* = .090, 95CI[-1.88,0.15]).

## 8 Discussion

This study examined the neural correlates of vicarious reward processing for peers who had previously engaged in including or excluding behaviors. In addition, we tested whether experiences of peer victimization averaged over two years were related to neural vicarious reward processing (interpretation of social information) and subsequent trust behaviors (behavioral response to social information) in late childhood. Three main findings emerged from the study. First, neural analyses revealed that the VS was more activated when winning compared to losing for oneself, but not when winning versus losing for others. VS activation did not differ between wins for self and includers compared to excluders (not supporting Hypothesis 1a), but losing for an excluder resulted in greater VS activation than losing for oneself (partially supporting Hypothesis 1b). VS activation was not predictive of subjective pleasure ratings of winning (not supporting Hypothesis 1c) and trust behaviors (not supporting Hypothesis 3). Additionally, the dmPFC, vmPFC, and precuneus showed increased activation when playing for another peer, compared with playing for oneself, highlighting the role of these brain regions in the social brain network (Blakemore, 2008, Blakemore, 2012). Second, neural activation in the VS and dmPFC was predicted by prior peer victimization experiences. Contrary to our hypotheses (2a and 2c), children with higher averaged peer victimization scores showed decreased VS activation when winning (versus losing) for themselves and when winning (versus losing) for themselves compared to winning (versus losing) for an excluder. In addition, children with higher averaged peer victimization scores showed increased dmPFC activation when playing for an excluder (versus playing for themselves), compared to children with lower peer victimization scores. Finally, peer victimization was related to subsequent trust behaviors, such that children with more victimization experiences were more discriminating in their trust behaviors toward includers and excluders (in contrast to Hypotheses 4a and 4b). These findings suggest that children with prior peer victimization experiences may show alterations in both the interpretation of and behavioral response to social information, such that they exhibit altered reward sensitivity and trust behaviors in specific contexts. Table S6 presents an overview of hypotheses, results and interpretations.

### 8.1 Neural correlates of vicarious reward processing

Consistent with previous studies highlighting the role of the VS in reward processing (Delgado, 2007, Haber and Knutson, 2010, Liu et al., 2011) and the role of the VS in affective processing and interpretation of a social stimulus (Nelson et al., 2005), whole-brain and ROI analyses revealed increased VS activation for winning compared to losing for oneself. In addition, VS activation was decreased when losing for oneself compared to losing for others, suggesting that losing money for oneself may be a particularly unrewarding experience. Interestingly, VS activation did not differ during wins and losses for others (either includers or excluders), consistent with the finding that the VS is more involved in personal reward processing compared to vicarious reward processing (Morelli et al., 2015). Although participants reported liking winning for the includer more than winning for the excluder and previous research has shown that the VS responds to vicarious rewards for familiar liked others (Braams et al., 2014, Braams et al., 2014), levels of VS activation during vicarious reward processing were similar in the includer and excluder conditions. Previous work has suggested that VS activation when winning for a friend is related to friendship quality (Braams et al., 2014, Schreuders et al., 2021) and that only participants who reported higher levels of closeness showed target-related differences in VS activation (Fareri et al., 2012a). It is possible that being included by a stranger, which may elicit positive feelings toward that person, does not create enough closeness to be distinguishable from the excluding stranger in terms of neural vicarious reward sensitivity (see Brandner et al., 2021). In addition, the includer and excluder may not have been close enough to the participant to elicit reward-related activation in the VS, which in turn may also explain the absence of a main effect of outcome (winning and losing) in the whole-brain analyses. Thus, our findings tentatively suggest that VS involvement in vicarious rewards for others is likely related to increased closeness and familiarity, rather than to positive feelings alone. To further explore these findings, future studies could compare the neural correlates of winning for a close other to winning for an unfamiliar includer. Another interesting direction for future research would be to examine how the formation of friendships (i.e., from strangers to friends) over time is related to changes in VS responses to vicarious rewards for that person.

In addition, whole-brain analyses revealed a strong network of activation in the dmPFC, vmPFC, and precuneus when playing for others compared to playing for oneself, regardless of the outcome (i.e., winning or losing). These findings aligned with the results of the ROI analyses and replicate previous work showing the involvement of the mPFC and precuneus when processing outcomes for peers versus oneself (Braams et al., 2014, Braams et al., 2014, Morelli et al., 2015). The mPFC and precuneus have often been implicated as key regions for other-focused processes such as perspective taking (Blakemore, 2008, Carrington and Bailey, 2009, Van Overwalle, 2009, Van Overwalle and Baetens, 2009) and have been linked to cognitive-regulatory processing of a social stimulus (Nelson et al., 2005). Furthermore, the differentiation of activation in the vmPFC and precuneus during vicarious versus personal rewards was previously found to be highest in late childhood/early adolescence and to become more similar with age (Braams and Crone, 2017a). ROI results additionally revealed a main effect of outcome in the dmPFC, such that dmPFC activation was higher during losses compared to wins. However, this effect was small and warrants replication in future studies. Contrary to our hypotheses and previous findings (Braams et al., 2014, Braams et al., 2014), we did not find increased activation in the TPJ when playing for others compared to playing for oneself. Given that activation in the TPJ during mentalizing increases throughout adolescence (Crone and Dahl, 2012) and was previously also not found during prosocial behavior in childhood (van der Meulen et al., 2018) these findings may suggest that the TPJ may become more involved in vicarious reward processing later in adolescence. Taken together, these findings point to increased neural activation related to mentalizing or perspective taking processes when winning or losing for others relative to oneself in late childhood.

### 8.2 Peer victimization, reward processing and trust

Based on the SIP model (Crick and Dodge, 1994), we expected that prior victimization experiences would affect both the interpretation of social information, referring to reward processing in a social context, and the response to social information, referring to trust in includers and excluders. Our second aim was to examine whether children who reported more peer victimization experiences over the previous two years would show altered neural activation during personal and vicarious reward processing. Victimization scores in the sample were relatively low, with few children reporting severe rates of victimization. Nevertheless, results indicated that children with higher victimization scores over the previous two years showed less VS activation when winning (vs. losing) for oneself, and less differentiation in VS activation when winning (vs. losing) for oneself compared to winning (vs. losing) for an excluder. These findings contradict those of an earlier study (Meuwese et al., 2018), which showed that adolescents who were less accepted by their peers showed more VS activation when winning money for themselves. In that study, peer acceptance was measured using peer acceptance and rejection (i.e., received nominations of being liked and disliked), which likely captures a different social process than longitudinal self-reports of peer victimization (e.g., Oldenburg et al., 2015). Peer victimization is a more negative experience, as it involves being the target of explicit hurtful behavior by peers. Potentially, the subjective experience of being victimized may have a greater impact on how social information is processed and interpreted compared to peer acceptance and rejection reported by others. Consistent with the findings of this study, relational peer victimization has been associated with a more blunted neural response to rewards in emerging adults (Ethridge et al., 2018). Our findings suggest that even less severe victims of bullying may show reduced neural sensitivity to personal monetary rewards compared to non-victims, as early as late childhood. Lower neural reward sensitivity has also been associated with an increased risk of adolescent depression (Keren et al., 2018, Luking et al., 2016), and exposure to bullying appears to predict later psychopathology, such as depression (Rijlaarsdam et al., 2021). Future studies are needed to examine how and whether altered affective processing of social information, such as lower reward sensitivity, in victimized children may predict long-term mental health outcomes (Oppenheimer et al., 2020, Palamarchuk and Vaillancourt, 2022) and the formation of healthy relationships, in order to advance our understanding of how best to support victimized children. In addition, victims of bullying have often been found to be more sensitive to social stimuli (Cubillo, 2022, Güroğlu and Veenstra, 2021) and more sensitive to social than to monetary rewards (Rappaport et al., 2019). We did not find a relation between victimization and increased neural sensitivity when winning for an includer or excluder, possibly suggesting that winning social rewards (i.e., receiving likes) and winning monetary rewards in a social context are separate processes.

In addition, results tentatively suggested that children with higher victimization scores over the previous two years showed greater dmPFC activation when playing for the excluder than when playing for themselves, regardless of the outcome. As a key region in the social brain network, the dmPFC has often been implicated in perspective taking and mentalizing (Andrews et al., 2021, Blakemore, 2008, Denny et al., 2012) and the cognitive regulation of emotions (Buhle et al., 2014, Silvers et al., 2017). Therefore, increased recruitment of the dmPFC when playing for others may possibly reflect increased thinking about others’ intentions or a greater need to control emotions when viewing the excluder. However, the effect was small, possibly due to the relatively small proportion of highly victimized children in the sample. Therefore, we emphasize the need to replicate the relation between dmPFC and victimization in future studies with more variation in victimization scores. Tentatively, these findings suggest that even relatively low levels of peer victimization in middle and late childhood may be associated with altered neural processing and interpretation of social information, in both affective neural regions such as the ventral striatum and cognitive-regulatory neural regions such as the dmPFC (see also Nelson et al., 2005).

Finally, children with more victimization experiences over the previous two years also differed in their subsequent trust toward the including and excluding peers (i.e., the behavioral response step of the SIP model). That is, more peer victimization was associated with greater differentiation in trust toward the includer and the excluder. Previous studies have shown inconsistent findings regarding trust and peer victimization, with some studies reporting lower generalized trust (Betts et al., 2017) and trust learning (Lenow et al., 2015), and others reporting higher trust beliefs toward peers (Rotenberg and Boulton, 2013) in children with higher peer victimization. Our findings show that the relation between victimization and trust may depend on the target of trust (see also van Noorden et al., 2016), such that victimized children may only show increased trust in peers who previously included them. This finding tentatively suggests that victimized children may place more importance on the social context and likeability of others when determining their behavioral response to others. Previous work has shown that victimized adolescent girls show increased attention and social monitoring toward in-group members, likely to satisfy their need to belong and gain acceptance within the in-group (Telzer et al., 2020). The present results extend these findings by showing that victimized children may also be more trusting of unknown, friendly peers. The greater differentiation in trust between liked and disliked others in victimized children may additionally suggest that victimized children are less likely to give disliked children a second chance, which in turn may likely limit their ability to establish positive relationships with others. Interestingly, neural sensitivity to rewards was did not predict subsequent trust. Future studies may further explore how and whether the interpretation of social information affects the decision for behavioral responses, by further examining the neural mechanisms underlying this differentiation in trust toward social partners.

### 8.3 Strengths and limitations

This study focused on the relation between neural correlates of vicarious reward processing and prior victimization experiences, thereby shedding light on the socio-cognitive processes underlying victimization in late childhood, a relatively understudied period in neuroimaging research. We used an innovative experimental task design in which participants performed a vicarious reward task for targets with whom they had previously interacted in a game of Cyberball. This allowed us to disentangle vicarious reward responses to liked and disliked strangers. In addition, victimization reports were collected in the two years prior to the study, providing longitudinal prospective data on victimization during the elementary school years, when victimization often occurs (Haltigan and Vaillancourt, 2014). Several limitations of the study should also be considered. First, most participants in the sample reported relatively low levels of victimization, which limits the generalizability of the findings to more severe victims of bullying. All participants were from schools that implemented the KiVa anti-bullying intervention, which may have led to lower rates of victimization in this sample, although prior work has shown that a subgroup remains victimized (Kaufman et al., 2018). Additionally, the recruitment of participants took place during the second lockdown of the Covid-19 pandemic in the Netherlands, which may have impacted the inclusion of families who experienced more stressors, such as severe victimization. Previous research has shown that families with more pre-existing stressors or challenges were more severely affected by the Covid-19 pandemic (Achterberg et al., 2021). Although the sample size of the study was larger than average for neuroimaging studies (Szucs and Ioannidis, 2020), future studies with larger sample sizes combined with more variation in victimization scores are needed to replicate the findings of this study. Second, trust behaviors were measured using a one-shot trust game in which participants received no feedback on the other player’s choices and did not interact with the other player again. However, in real life, it is likely that trust occurs in social interactions where trust reciprocity may influence future trust decisions and this updating of trust beliefs may be influenced by past experiences (Fareri et al., 2012b). An interesting direction for future research would be to use multiple-round trust games to assess whether children with a history of peer victimization also show altered updating of trust beliefs and behaviors, potentially affecting their ability to form social relationships. Finally, there may be several mediating or confounding effects that may explain the relation between victimization experiences and altered neural processing of rewards. For example, both victimization and reward sensitivity have been linked to parental influences (Kokkinos, 2013, Telzer et al., 2015), loneliness (Dziura et al., 2023, Matthews et al., 2022) and depression (Luking et al., 2016, Rijlaarsdam et al., 2021). In addition, although data were collected after the initial peak of the Covid-19 pandemic and not during periods of lockdown or school closure, the Covid-19 pandemic may still have affected social developmental processes and mental health in victims of bullying. For example, lower peer connectedness combined with lower reward sensitivity was associated with suicidal ideation and depressive symptoms during Covid-19 (Hutchinson et al., 2021, Sequeira et al., 2021). Future research with larger sample sizes is needed to further examine the possible explanatory mechanisms underlying the altered neural reward sensitivity and trust behaviors in victims of bullying, and the implications for long-term mental health.

## 9 Conclusion

In conclusion, our findings demonstrate the involvement of the VS in processing personal rewards and of the dmPFC, vmPFC, and precuneus in processing and interpreting losses and rewards for others. Victimization was related to decreased VS activation when receiving rewards for oneself, suggesting that victimized children may have lower personal reward sensitivity. In addition, victimization was related to increased dmPFC activation when processing losses and rewards for excluders compared to oneself, possibly indicating greater involvement of mentalizing or emotional control processes during vicarious reward processing. Finally, children with more victimization over the previous two years showed a greater differentiation in subsequent trust toward including and excluding peers, highlighting an increased importance of interaction partners in trust decisions for children with prior victimization experiences. Taken together, these findings help shed light on the social cognitions and behaviors of victims of bullying and may help identify the mechanism by which peer victimization may affect adolescents’ ability to form and maintain healthy relationships, a core developmental task of adolescence.

## CRediT authorship contribution statement

**René Veenstra:** Writing – review & editing, Funding acquisition, Conceptualization. **Sanne Kellij:** Writing – review & editing, Project administration, Investigation, Data curation, Conceptualization. **Simone Dobbelaar:** Writing – review & editing, Writing – original draft, Visualization, Formal analysis, Data curation, Conceptualization. **Berna Güroğlu:** Writing – review & editing, Supervision, Funding acquisition, Conceptualization.

## Declaration of Competing Interest

The authors declare that they have no known competing financial interests or personal relationships that could have appeared to influence the work reported in this paper.

## Data Availability

Data will be made available on request.
