# Supplementary material for: Neural correlates of vicarious reward processing and peer victimization experiences in late childhood
Source: Dev Cogn Neurosci. 2024 Dec 24;71:101499. doi: 10.1016/j.dcn.2024.101499 (PMC11743848; doi:10.1016/j.dcn.2024.101499)
Supplement: Tables S1 — Supplementary material [file mmc1.docx]

**Supplementary Results**

***Whole-brain analyses***

To examine the neural correlates of vicarious reward processing, we performed a repeated measures ANOVA with the within-person factors ‘target’ (three levels: self, includer, excluder) and ‘outcome’ (two levels: win, lose) in SPM12. This preregistered whole-brain analysis revealed a main effect of target, with activation in the precuneus, dorsomedial prefrontal cortex and ventromedial prefrontal cortex (see Figure S1a and Table S1 for the MNI coordinates of clusters of activation). Follow-up pairwise contrasts revealed that these regions were increasingly activated during the includer and excluder conditions compared to the self condition (see Table S1). There was no main effect of outcome and no interaction effect of target and outcome.


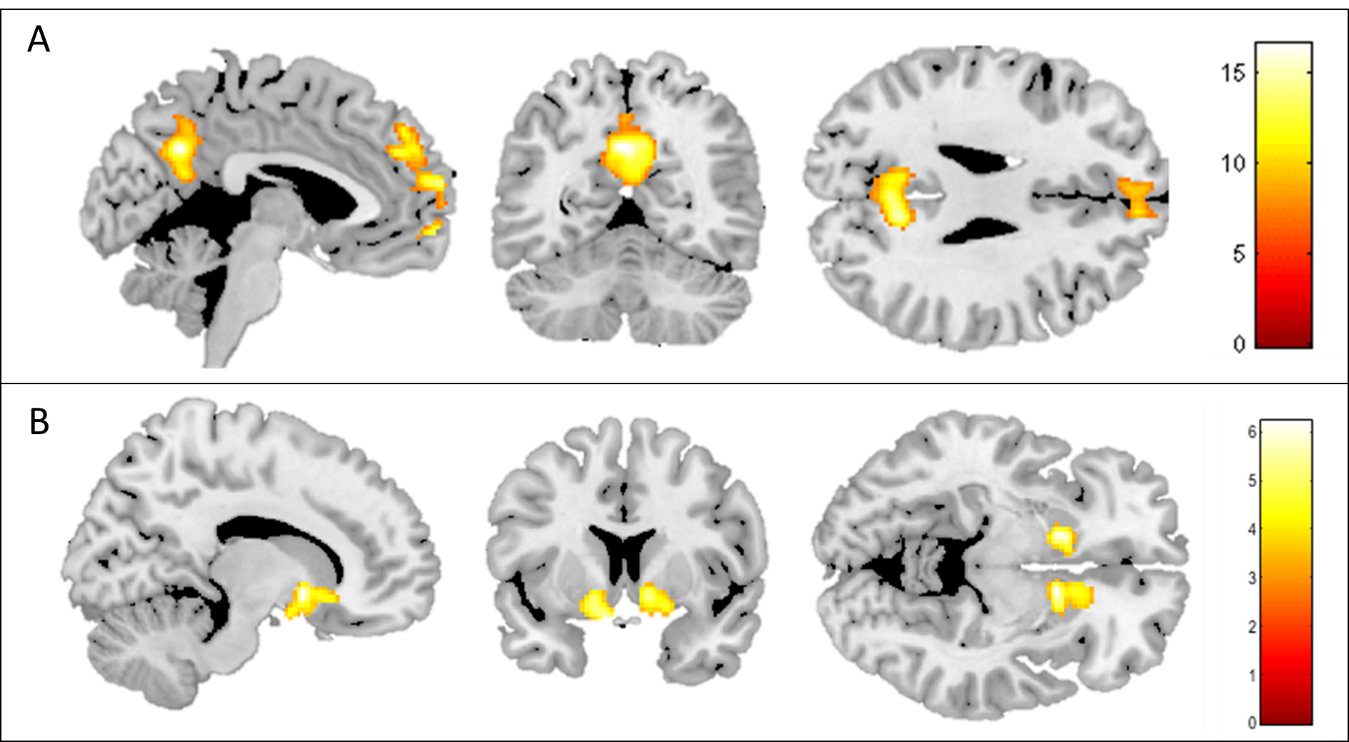
The separate preregistered contrast “SelfWin > SelfLose” resulted in activation in the right putamen and left caudate nucleus (see Figure S1b and Table S2). The contrasts “IncluderWin > IncluderLose”, and “ExcluderWin > ExcluderLose” did not reveal significant clusters of activation.

Figure S1. A) Whole-brain activation (MNI coordinates (*x,y,z*): -4,-58,28) in the main effect of target (three levels: self, includer, excluder) of the repeated measures ANOVA with within-person factors of target and outcome. B) Whole-brain activation in the contrast “SelfWin > SelfLose”. Results were FWE cluster-level corrected (P_FWEcc_<.05).


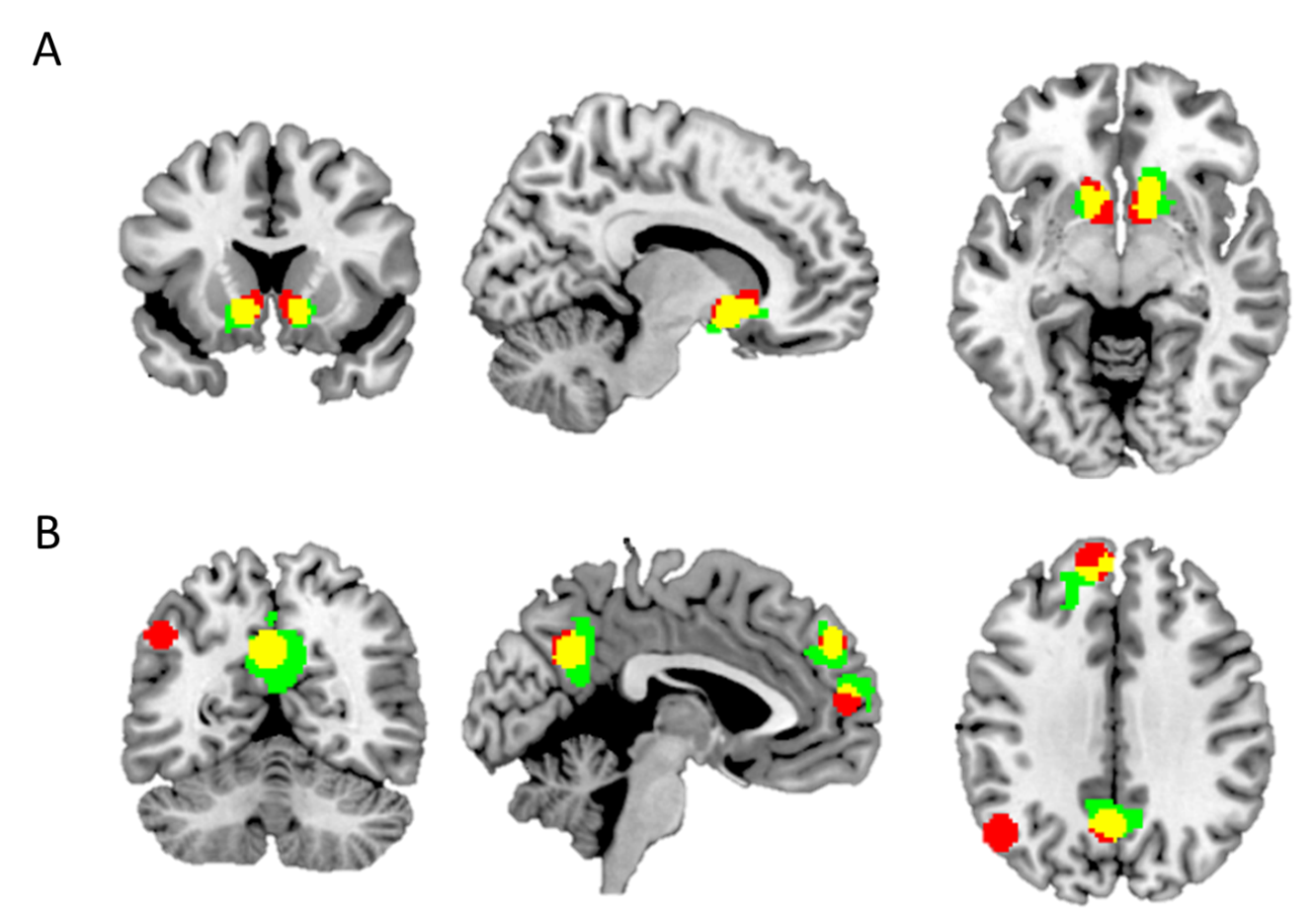


Figure S2. Overlap between whole-brain activation and independent regions of interest. A) Whole-brain activation in the contrast “SelfWin > SelfLose” (in green), independent anatomical ROI of the ventral striatum (in red) and overlap of activation (in yellow). B) Whole-brain activation in the main effect of target in the repeated measures ANOVA (in green), independent ROIs of dmPFC, vmPFC, precuneus and TPJ (in red) and overlap of activation (in yellow).

Table S1. MNI coordinates for local maxima activation in the regions identified in the repeated measures ANOVA, in the main effects of Target (three levels: self, includer, excluder), Outcome (two levels: win, lose) and the interaction of Target × Outcome. For the main effect of Target, post-hoc pairwise contrasts of Includer > Self and Excluder > Self are reported.

| **Anatomical region** | **Voxels** | ***pFWEcc*** | ***F / T*** | **MNI coordinates** | | |
| --- | --- | --- | --- | --- | --- | --- |
|  |  |  |  | ***x*** | ***y*** | ***z*** |
| ***Main effect of target*** | | | | | | |
| Left precuneus | 1018 | < .001 | 16.59 | -2 | -58 | 32 |
|  |  |  | 9.86 | -2 | -54 | 44 |
| Right precuneus |  |  | 15.12 | 8 | -54 | 28 |
| Left superior medial gyrus | 1161 | < .001 | 16.26 | -6 | 66 | 16 |
| Right superior medial gyrus |  |  | 13.20 | 8 | 64 | 14 |
|  |  |  | 12.89 | -6 | 44 | 32 |
|  |  |  |  |  |  |  |
| *Includer > Self* |  |  |  |  |  |  |
| Left superior medial gyrus | 1614 | < .001 | 5.41 | -6 | 66 | 16 |
| Right superior medial gyrus |  |  | 4.91 | 8 | 64 | 14 |
| Left superior frontal gyrus |  |  | 4.72 | -18 | 34 | 36 |
| Right angular gyrus | 289 | .043 | 5.27 | 56 | -64 | 26 |
| Left precuneus | 1194 | < .001 | 5.27 | -4 | -58 | 34 |
|  |  |  | 3.92 | -4 | -54 | 42 |
| Right precuneus |  |  | 5.15 | 8 | -54 | 28 |
| Left precuneus | 295 | .041 | 5.03 | -12 | 14 | -10 |
| Left amygdala |  |  | 4.14 | -12 | 2 | -16 |
| Right olfactory cortex |  |  | 4.09 | 4 | 14 | -12 |
|  |  |  |  |  |  |  |
| *Excluder > Self* |  |  |  |  |  |  |
| Left superior frontal gyrus | 1789 | < .001 | 5.23 | -12 | 40 | 52 |
|  |  |  | 4.58 | -10 | 32 | 54 |
|  |  |  | 4.52 | -2 | 60 | 18 |
| Left precuneus | 1166 | < .001 | 4.78 | -2 | -58 | 32 |
| Right precuneus |  |  | 4.57 | 6 | -56 | 32 |
|  |  |  | 4.47 | 4 | -56 | 20 |
|  |  |  |  |  |  |  |
| ***Main effect of outcome*** | | | | | | |
| n.s. |  |  |  |  |  |  |
|  |  |  |  |  |  |  |
| ***Interaction effect of target × outcome*** | | | | | | |
| n.s. |  |  |  |  |  |  |

*Note.* Results were cluster-level corrected at *p*_FWEcc_ < .05, with a primary threshold of *p*<.001 and *k*=10. n.s. = no significant clusters of activation.

Table S2. MNI coordinates for local maxima activation in the regions identified in the contrast “SelfWin > SelfLose”

| **Anatomical region** | **Voxels** | ***pFWEcc*** | ***T*** | **MNI coordinates** | | |
| --- | --- | --- | --- | --- | --- | --- |
|  |  |  |  | ***x*** | ***y*** | ***z*** |
| ***SelfWin > SelfLose*** | | | | | | |
| Left parahippocampal gyrus | 307 | .017 | 6.22 | -14 | 4 | -14 |
| Left putamen |  |  | 5.96 | -14 | 12 | -8 |
| Right caudate nucleus | 347 | .010 | 6.21 | 12 | 10 | -6 |
|  |  |  | 4.45 | 10 | 18 | -8 |
|  |  |  | 4.31 | 12 | 4 | -16 |

*Note.* Results were cluster-level corrected at *p*_FWEcc_ < .05, with a primary threshold of *p*<.001 and *k*=10. n.s. = no significant clusters of activation.

Table S3. Statistics of the repeated measures ANOVAs with within-person effects of Target (three levels: self, includer, excluder), outcome (two levels: win, lose), and the interaction of Target × Outcome on neural activation in the five ROIs (VS, vmPFC, dmPFC, precuneus, TPJ).

|  | ***F*** | ***df*** | ***p (unc.)*** | ***p (B-H corr.)*** | ***η^2^*** |
| --- | --- | --- | --- | --- | --- |
| ***VS*** |  |  |  |  |  |
| Target | 8.43 | 1.77, 107.86 | <.001 | .004 | .04 |
| Outcome | 6.81 | 1, 61 | .011 | .034 | .01 |
| Target × Outcome | 12.36 | 2, 122 | <.001 | .001 | .03 |
|  |  |  |  |  |  |
| ***vmPFC*** |  |  |  |  |  |
| Target | 7.01 | 2, 122 | .001 | .005 | .04 |
| Outcome | 1.02 | 1, 61 | .317 | .510 | <.001 |
| Target × Outcome | 3.23 | 2, 122 | .043 | .106 | .01 |
|  |  |  |  |  |  |
| ***dmPFC*** |  |  |  |  |  |
| Target | 19.52 | 1.82, 111.03 | <.001 | <.001 | .08 |
| Outcome | 5.87 | 1, 61 | .018 | .048 | .01 |
| Target × Outcome | 0.16 | 2, 122 | .850 | .925 | <.001 |
|  |  |  |  |  |  |
| ***Precuneus*** |  |  |  |  |  |
| Target | 14.69 | 2, 122 | <.001 | <.001 | .06 |
| Outcome | 0.21 | 1, 61 | .646 | .747 | <.001 |
| Target × Outcome | 0.98 | 2, 122 | .379 | .571 | .01 |
|  |  |  |  |  |  |
| ***TPJ*** |  |  |  |  |  |
| Target | 2.74 | 1.81, 110.21 | .075 | .163 | .01 |
| Outcome | 0.41 | 1, 61 | .525 | .627 | <.001 |
| Target × Outcome | 0.10 | 2, 122 | .904 | .929 | <.001 |

*Note.* Abbreviations: unc. = uncorrected, B-H corr. = Benjamini-Hochberg corrected, VS = ventral striatum, vmPFC = ventromedial prefrontal cortex, dmPFC = dorsomedial prefrontal cortex, TPJ = temporal parietal junction.

Table S4. Statistics of the linear regressions with averaged victimization as predictor and neural ROI activation as outcome variable.

| ***Neural contrast*** | ***t*** | ***df*** | ***p (unc.)*** | ***p (B-H corr.)*** | ***β*** |
| --- | --- | --- | --- | --- | --- |
| ***VS*** |  |  |  |  |  |
| SelfWin – SelfLose | -2.82 | 60 | .007 | .022 | -0.34 |
| ExclWin – ExclLose | 0.87 | 60 | .386 | .571 | 0.11 |
| InclWin – InclLose | -1.35 | 60 | .138 | .269 | -0.17 |
| (SelfWin – SelfLose) – (ExclWin – ExclLose) | -2.88 | 60 | .006 | .020 | -0.35 |
| (InclWin – InclLose) – (ExclWin – ExclLose) | -1.71 | 60 | .093 | .192 | -0.17 |
|  |  |  |  |  |  |
| ***vmPFC*** |  |  |  |  |  |
| Excl – Self | 1.41 | 60 | .165 | .291 | 0.17 |
| Incl – Self | 0.65 | 60 | .519 | .627 | 0.08 |
|  |  |  |  |  |  |
| ***dmPFC*** |  |  |  |  |  |
| Excl – Self | 2.46 | 60 | .017 | .048 | 0.30 |
| Incl – Self | 1.95 | 60 | .056 | .130 | 0.24 |
| Lose – Win | 1.05 | 60 | .297 | .500 | 0.13 |
|  |  |  |  |  |  |
| ***Precuneus*** |  |  |  |  |  |
| Excl – Self | -0.08 | 60 | .936 | .936 | -0.01 |
| Incl – Self | -0.75 | 60 | .456 | .625 | -0.10 |

*Note.* Abbreviations: unc. = uncorrected, B-H corr. = Benjamini-Hochberg corrected, VS = ventral striatum, vmPFC = ventromedial prefrontal cortex, dmPFC = dorsomedial prefrontal cortex.

Table S5. Statistics of the exploratory non-preregistered linear regressions with averaged victimization as predictor and ROI activation in the vmPFC, dmPFC, precuneus and TPJ in the contrasts “SelfWin-SelfLose”, “IncluderWin-IncluderLose”, “ExcluderWin-ExcluderLose” as outcome variable.

| ***Neural contrast*** | ***t*** | ***df*** | ***p*** | ***β*** |
| --- | --- | --- | --- | --- |
| ***vmPFC*** |  |  |  |  |
| SelfWin – SelfLose | 0.89 | 60 | .376 | 0.11 |
| InclWin – InclLose | -0.99 | 60 | .327 | -0.13 |
| ExclWin – ExclLose | 0.32 | 60 | .750 | 0.04 |
|  |  |  |  |  |
| ***dmPFC*** |  |  |  |  |
| SelfWin – SelfLose | 0.97 | 60 | .334 | 0.12 |
| InclWin – InclLose | -0.80 | 60 | .430 | -0.10 |
| ExclWin – ExclLose | -0.93 | 60 | .356 | -0.12 |
|  |  |  |  |  |
| ***Precuneus*** |  |  |  |  |
| SelfWin – SelfLose | -0.68 | 60 | .498 | -0.09 |
| InclWin – InclLose | -1.30 | 60 | .197 | -0.17 |
| ExclWin – ExclLose | -1.26 | 60 | .214 | -0.16 |
|  |  |  |  |  |
| ***TPJ*** |  |  |  |  |
| SelfWin – SelfLose | 0.53 | 60 | .599 | 0.07 |
| InclWin – InclLose | -2.03 | 60 | .047 | -0.25 |
| ExclWin – ExclLose | -0.66 | 60 | .511 | -0.08 |

*Note.* Abbreviations: vmPFC = ventromedial prefrontal cortex, dmPFC = dorsomedial prefrontal cortex, TPJ = temporal parietal junction.

Table S6. Overview of hypotheses, step in the Social Information Processing model, results and interpretations / implications.

|  | Hypothesis | SIP model step | Result | Interpretation / Implication |
| --- | --- | --- | --- | --- |
| 1a | Greater VS activation when winning for self / includer vs. winning for excluder | Interpretation | Greater VS activation when winning vs. losing for oneself, but no difference between VS activation when winning for includer and excluder | - VS mainly involved in personal reward processing compared to vicarious reward processing - VS involvement in vicarious rewards possibly related to closeness or familiarity, instead of positive feelings alone |
| 1b | Greater VS activation when losing for excluder vs. losing for self or includer | Interpretation | Greater VS activation when losing for excluder vs. losing for oneself |  |
| 1c | Greater VS activation when winning vs. losing for includer / excluder 🡪 Greater self-reported pleasure when winning for includer / excluder | Interpretation | VS activation did not predict self-reported pleasure |  |
| 2a | More victimization 🡪 more VS activation when winning (vs. losing) for oneself | Interpretation | More victimization 🡪 *less* VS activation when winning for self | - Even less severe victims of bullying may show blunted neural sensitivity for personal rewards already in late childhood - Lower neural reward sensitivity may be related to increased risk for depression |
| 2b | More victimization 🡪 less VS activation when winning (vs. losing) for excluder | Interpretation | Victimization did not predict VS activation when winning (vs. losing) for an excluder |  |
| 2c | More victimization 🡪 more VS activation when winning (vs. losing) for oneself versus winning (vs. losing) for excluder | Interpretation | More victimization 🡪 *less* VS activation when winning (vs. losing) for oneself versus winning (vs. losing) for excluder |  |
| - | *Secondary analysis* | Interpretation | More victimization 🡪 more dmPFC activation when playing for excluder vs. self | - Victims of bullying may possibly mentalize more or need more emotion regulation when interacting with a disliked peer |
| 3 | More VS activation when winning for includer/excluder 🡪 more trust toward includer/excluder | Interpretation 🡪 Behavioral response | VS activation did not predict trust behavior | - No direct relation between neural reward sensitivity and trust |
| 4a | More trust toward includer vs. excluder | Behavioral response | No difference in trust toward includer and excluder | - Relation between victimization and trust depend on target - Victimized children may be less likely to give disliked peers second chance |
| 4b | More victimization 🡪 less trust (regardless of target) | Behavioral response | More victimization 🡪 more differentiation in trust toward excluder and includer |  |
